# Triple-Loaded Nanoemulsions Incorporating Coffee Extract for the Photoprotection of Curcumin and Capsaicin: Experimental and Computational Evaluation

**DOI:** 10.3390/pharmaceutics17070926

**Published:** 2025-07-17

**Authors:** Nuttapol Boonrueang, Siripat Chaichit, Wipawadee Yooin, Siriporn Okonogi, Kanokwan Kiattisin, Chadarat Ampasavate

**Affiliations:** 1 Department of Pharmaceutical Sciences, Faculty of Pharmacy, Chiang Mai University, Chiang Mai 50200, Thailand; nuttapol_b@cmu.ac.th (N.B.); siripat.chaichit@cmu.ac.th (S.C.); wipawadee.y@cmu.ac.th (W.Y.); siriporn.okonogi@cmu.ac.th (S.O.); kanokwan.k@cmu.ac.th (K.K.); 2 Center of Excellence in Pharmaceutical Nanotechnology, Faculty of Pharmacy, Chiang Mai University, Chiang Mai 50200, Thailand

**Keywords:** photostability, turmeric, chili, coffee, nanoemulsions, interaction, stabilization

## Abstract

**Background/Objectives**: This study aims to present a strategic approach to enhancing the photostability and antioxidative resilience of curcumin and capsaicin by integrating selected natural stabilizers within a nanoemulsion-based delivery system. **Methods**: Coffee extract (*Coffea arabica* Linn.), along with its active components and vitamin E-containing natural oils, was assessed in terms of improving the photostabilizing and antioxidative retention abilities of curcumin and capsaicin. An optimized ratio of the active mixture was then loaded into a nanoformulation. **Results**: The analysis of active contents with validated high-performance liquid chromatography (HPLC), ferric reducing antioxidant power (FRAP), and 2,2-diphenyl-1-picrylhydrazyl (DPPH) assays confirmed the stabilization enhancement after irradiation with UV and white light for 72,000–84,000 lux hours. The optimized combination of coffee extract with turmeric and chili mixtures loaded into the optimized nanoemulsion enhanced the half-lives (T_1/2_) of curcumin and capsaicin by 416% and 390%, respectively. The interactions of curcumin and capsaicin with caffeine and chlorogenic acid were elucidated using computational calculations. Interaction energies (E_int_), HOMO-LUMO energy gap (HLG) analysis, and global reactivity descriptors revealed hydrogen bonding interactions be-tween capsaicin and chlorogenic acid, as well as between curcumin and caffeine. **Conclusions**: By leveraging the synergistic antioxidative properties of coffee extract and vitamin E within a nanoemulsion matrix, this study overcomes the intrinsic stability limitations of curcumin and capsaicin, offering a robust platform for future pharmaceutical and nutraceutical applications.

## 1. Introduction

Turmeric and chili, popular spices in South and Southeast Asian countries, have shown promise in addressing many diseases, especially those rooted in inflammation. Curcumin in turmeric (*Curcuma longa* Linn., family Zingiberaceae) and capsaicin in chili (*Capsicum chinense*, family Solanaceae) are pharmacologically active compounds present in these common spices, which are documented in the pharmacopoeias of many countries [1,2]. However, their limited water solubility and stability, especially upon light exposure, impedes their bioavailability and in vivo applications. Turmeric’s rhizome contains important chemical constituents, known as curcuminoids, which are yellow pigments comprising curcumin, demethoxycurcumin, and bis-demethoxycurcumin. Turmeric is often referred to as the ‘Golden Spice’ or ‘Indian Solid Gold’ [3]. On the other hand, chili contains important chemical components known as capsaicinoids [4]. Over 20,000 articles on curcumin or capsaicin have been published in the past decade [5]. Both compounds have demonstrated anti-inflammatory, antioxidant, and anti-cancer properties. Focusing on their anti-inflammatory activity, the constitutive and inducible cyclooxygenase enzymes (COX-1 and COX-2) and IκB-α degradation are inhibited by both compounds, thereby preventing the production of eicosanoid prostaglandin E2, 5-hydroxyeicosatetraenoic acid, and cytokines from the NF-κB signaling pathway [6,7,8]. However, limited pharmaceutical formulations and indications related to these plants have been marketed. This could be due to the major physicochemical drawbacks of their active compounds. The efficacy of curcumin and capsaicin is limited by their poor stability, especially photostability, poor aqueous solubility, and very low oral bioavailability (less than 1%) [9,10]. In the presence of water, curcumin is rapidly degraded by oxidation and hydrolysis reactions. The half-life of curcumin in aqueous buffer at physiological pH is only a few minutes, causing the generation of various degraded products, such as ferulic acid, feruloyl methane, and the recently discovered bicyclopentadione derivatives of curcumin [11,12,13]. Capsaicin and dihydrocapsaicin were relatively stable under gamma irradiation up to 15 kGy [14]. However, water-soluble pigments and capsanthin were degraded, whereas capsaicin remained stable as the electron beam irradiation dose increased [15].

Three major approaches to enhancing the stability of light-sensitive compounds have been revealed: (1) adding antioxidants, such as lecithin, quercetin, genistein, eugenol, terpinol, etc.; (2) novel drug delivery platforms, such as nanoparticles, liposomes, micelles, and phospholipid complexes; and (3) adding photoprotective agents [16,17,18,19]. Recently, the photostabilization of curcumin and capsaicin was reported by our group using a potent antioxidant coffee derivative, chlorogenic acid, as a natural stabilizer entrapped in nanostructure lipid carriers (NLCs) [20]. However, the stabilizing efficiency of coffee extracts and their isolated components for curcumin and capsaicin was not compared. In addition, a stability enhancement, along with other advantages such as attractive appearance, sensorial benefits, and enhanced safety issues, was reported when curcumin was formulated in nanoemul-sions [21,22]. Therefore, in the present study, attempts to maximize the stability and antioxidative activity of curcumin and capsaicin in turmeric and chili extracts formulated as a nanoemulsion were systematically investigated. The approach included fortification with natural stabilizers, namely roasted Arabica coffee bean extract (*Coffea arabica* Linn.), vitamin E (DL-α-tocopheryl acetate), and avocado oil. A forced photodegradation protocol, conducted in accordance with a critical assessment of the ICH guideline on photostability testing [23], was applied to evaluate the stabilizing efficiency of the systems. The molecular interactions of selected combinations were further investigated between active compounds and stabilizers through Density Functional Theory (DFT) simulations. The appropriate development of a photostability-indicating assay method for the simultaneous determination of curcumin, capsaicin, and caffeine in alcoholic solutions was also presented.

## 2. Materials and Methods

### 2.1. Plant Materials

Turmeric was purchased from Specialty Natural Products (Chonburi, Thailand). An ethanolic chili extract was kindly provided by Bangkok Lab and Cosmetic (Ratchaburi, Thailand). Both extracts were received with certificates of analysis (CoAs) from accredited laboratories, and they were used as is. Roasted Arabica coffee beans were purchased from Doi Chang Coffee Original, (Bangkok, Thailand). The coffee beans were ground and dried in a hot air oven at 50 ± 2 °C for 24 h. The ground beans were then macerated with 95% ethanol (*v*/*v*) for 48 h for 3 cycles. After filtration through a Whatman^®^ No.1 filter paper (GE Healthcare, Buckinghamshire, UK), the solvent was evaporated to dryness using a rotary evaporator. The resulting extract was stored in amber glass containers and kept in a refrigerator. The contents of curcumin, capsaicin, and caffeine in the turmeric, chili, and coffee extracts, respectively, were repeatedly quantified by comparison with standards using validated high-performance liquid chromatography (HPLC) analysis. The turmeric extract contained 15.50 ± 0.03 g curcumin per 100 g extract, the ethanolic chili extract contained 6.04 ± 0.12 g capsaicin per 100 g extract, and the coffee extract contained 6.62 ± 0.03 g caffeine per 100 g extract. Although the coffee extraction was performed in-house, no additional purification steps were applied after extraction.

### 2.2. Chemical and Reagents

The capsaicin (98.94%) certified working standard was from Himedia (Mumbai, India). Curcumin (98.79%), 2,2-diphenyl-1-picrylhydrazyl (DPPH), caffeine (99.4%), chlorogenic acid (95.0%), and dimethyl sulfoxide (DMSO) were purchased from Sigma-Aldrich (St. Louis, MO, USA). Methyl red was purchased from Merck (Darmstadt, Germany). Polyacrylamide (and) C13-14 Isoparaffin (and) Laureth-7 were purchased from Seppic (Castres, France). Polyglyceryl-3 rice branate was purchased from Sinerga Spa (Varese, Italy). 2,4,6-Tripyridyl-s-triazine (TPTZ) was purchased from Fluka (Buchs, Switzerland). Acetonitrile, methanol, ethanol (HPLC grade), glacial acetic acid, ethanol (AR grade), and deionized water were purchased from RCI Labscan (Bangkok, Thailand). Tween 80 was purchased from NOF Corporation (Tokyo, Japan). Acetate buffer and ferric chloride (FeCl_3_) were purchased from LOBA Chemie (Mumbai, India). Distilled water and deionized (D.I.) water were used for the formulation and HPLC analysis, respectively.

### 2.3. Identification of Active Compound in Coffee Extract

The active compounds in coffee extracts were analyzed using HPLC, according to the method reported by Jandang et al. [20]. Suitable conditions for active compound determination in the coffee extract were achieved using a C18 analytical column (KNAUER^®^, Vertex III, 4.6 mm × 250 mm; 5 μm particle size (Knauer Wissenschaftliche Geräte GmbH, Berlin, Germany). Gradient elution was performed with a mobile phase of acetonitrile and 1% acetic acid in water (15:85), at a flow rate of 1.0 mL/min. Sample injections (10 µL) were detected at 280 nm. Standard stock solutions of caffeine and chlorogenic acid (1000 µg/mL in methanol) were serially diluted with a 50:50 methanol–water mixture to obtain working concentrations of 10–120 µg/mL. The extract of coffee was dissolved in ethanol at 5000 µg/mL and analyzed under the same conditions.

### 2.4. Mixtures of Extract Solutions for Photo- and Antioxidative Stability Tests

To verify the photostability and antioxidative stability of curcumin and capsaicin, the extract solutions were prepared with and without selected stabilizers such as coffee extract, tocopherol acetate (vitamin E), and avocado oil. Extracts of turmeric, chili, and coffee were accurately weighed to contain an equal amount of the corresponding active marker and diluted with ethanol to contain total active markers of 2000 μg/mL. Vitamin E and avocado oil were also diluted in Tween 80 and ethanol (4:6) to yield a concentration of 2000 μg/mL. Each extract and stabilizer(s) were pipetted to a 10 mL volumetric flask, containing an equal total concentration of active substances of 50 μg/mL, and were dissolved with ethanol before subjecting to the photostability study.

### 2.5. Preparation of Extract-Loaded Nanoemulsions

#### 2.5.1. Solubility Study of the Extracts in Various Vehicles

Each extract (50 mg) was placed in a microcentrifuge tube, and the test vehicles (avocado oil, dimethyl sulfoxide (DMSO), ethanol, methanol, glycerin, polyethylene glycol 400 (PEG 400), propylene glycol (PG), 10% *v*/*v* Tween 80, and distilled water) were pipetted incrementally in 50 μL aliquots, with vortex mixing after each addition to ensure proper dispersion, until the extract was completely dissolved. The mixtures were thoroughly blended using a vortex mixer after each addition. Solubility was visually assessed and evaluated based on the criteria outlined in the British Pharmacopoeia [24].

#### 2.5.2. Development of Turmeric, Chili, and Coffee Extract-Loaded Nanoemulsions

The development of nanoemulsions was carried out through three main steps, including the selection of pre-emulsion compositions, optimization of high-pressure homogenization, and preparation of extract-loaded nanoemulsions. Oil-in-water (O/W) nanoemulsions were formulated due to their increasing favor among consumers, who appreciate their unique properties, such as thermodynamic stability and the ability to form isotropic dispersions for active compounds with both lipophilic (curcumin and capsaicin) and hydrophilic (caffeine) properties. In addition, O/W nanoemulsions are preferred in cosmetics due to their fluidity, smooth feel on the skin, and ease of spreading [25]. The oil-in-water pre-emulsion system was formulated with a fixed oil phase constituting 21% *w*/*w* of the total formulation. The oil phase consisted of avocado oil (10%), rice bran oil (5%), mineral oil (5%), and DL-α-tocopheryl acetate (1%), while the aqueous phase contained PEG 400 (5%), phenoxyethanol (1%), and purified water, as shown in Table 1. Emulsifying agents, including polyacrylamide (and) C13-14 isoparaffin (and) laureth-7 and polyglyceryl-3 rice branate, were evaluated at concentrations ranging from 1% to 6%, with detailed results provided in Supplementary Material S1. Both the oil and water phases were preheated to 75 °C and 80 °C, respectively, before passing to homogenization using a high-shear homogenizer (IKA^®^ T25 digital Ultra-Turrax, Staufen, Germany) at 8400 rpm for 10 min to form the pre-emulsions. The initial physical stability of the pre-emulsions was assessed based on phase separation after 24 h. Subsequently, the pre-emulsions were further processed using a high-pressure homogenizer (APV 1000, SPX FLOW, Charlotte, NC, USA) at 500 bar for 5, 10, or 15 min. Optimal homogenization conditions were determined based on efficient droplet size reduction without compromising thermal stability. To prepare extract-loaded nanoemulsions, turmeric and chili extracts were dissolved in Tween 80 and incorporated into the oil phase. The coffee extract was solubilized in Tween 80 and added to the aqueous phase, as detailed in Table 2. The resulting nanoemulsions were evaluated for physical appearance, pH, particle size, polydispersity index (PDI), and zeta potential. Each formulation batch (100 g) was prepared in triplicate to ensure the reproducibility and reliability of the results.

#### 2.5.3. Characteristics and Stability of Extract-Loaded Nanoemulsions

Extract-loaded nanoemulsions were assessed for their mean internal droplet size and polydispersity index (PDI) using Zetasizer^®^ Nano Zs (Malvern, Worcestershire, UK), and their zeta potential was determined using the same instrument equipped with Laser Doppler Anemometry (LDA)/phase analysis light scattering (PALS). The formulation was diluted at a ratio of 1:100 with deionized water. The condition of Zetasizer^®^ was set at a room temperature (25 ± 0.5 °C) with a detection angle of 173°. The measurement was performed in triplicate. The physical stability of the extract-loaded nanoemulsion kept in amber and tight containers was examined as a function of temperature and time at 4 °C and 30 °C for 2 months. The hydrodynamic diameter, polydispersity value, and zeta potential value at the initial time point and at 30 and 60 days were monitored for the effects of storage conditions.

#### 2.5.4. Determination of Percentage Entrapment Efficiency

The entrapment efficiency of extract-loaded nanoemulsions was analyzed using ultrafiltration with some modifications. The sample was divided into 2 parts. In the first part, one milliliter of extract-loaded nanoemulsions was weighed and mixed with 5 mL of ethanol as an extracting solvent. The sample was vortexed for 1 min and left to sit in ethanol for 30 min before being subjected to the centrifugation at 10,000 rpm (16,993× *g*) at 25 °C for 10 min (Thermo Fisher Scientific, Sorvall ST16R, Merck, Darmstadt, Germany). The supernatant was collected and analyzed by HPLC for total curcumin, capsaicin, and caffeine [W_total_]. In the second part, one milliliter of extract-loaded nanoemulsions was weighed and added into the inner part of the ultrafiltration (Amicon^®^ Ultra-1.5 mL, NMWCO 10 kDa, Merck, Darmstadt, Germany). After that, it was centrifuged at 7000 rpm (11,895× *g*) at 25 °C for 1 h [26]. The unloaded extract solution was dropped into the bottom of the ultrafiltration tube [W_free_]. Aliquots of the supernatant [W_total_] and the filtrate [W_free_] were diluted with ethanol and mixed with 500 µL of methyl red solution (IS) at a ratio of 1:1. All samples were analyzed for active components at 425 and 280 nm using HPLC-DAD. Finally, the percentage of entrapment efficacy was calculated by the following equation:

%EE = (W_total_ − W_free_/W_total_) × 100 (1)

where W_total_ is the amount of total curcumin, capsaicin, and caffeine in the formulation and W_free_ is the amount of untrapped curcumin, capsaicin, and caffeine in the formulation.

### 2.6. Determination of Major Markers in Turmeric, Chili, and Coffee Extracts

To assess the stability of curcumin and capsaicin, the stability-indicating analytical method using reversed-phase high-performance liquid chromatography (RP-HPLC) with UV detection was developed and validated based on our previous study with slight modification [27]. The photo- and antioxidative stability of curcumin and capsaicin with and without stabilizers were evaluated upon exposure to UV and white light in a photostability testing chamber for 0, 4, 8 and 12 h.

#### Chromatographic System

The analytical method was adapted from a previously published study [27]. HPLC analysis was conducted using an Agilent 1260 Infinity system (Santa Clara, CA, USA), consisting of a mobile phase reservoir, quaternary pump, autosampler, thermostatted column compartment, and diode array detector. A Purospher^®^ STAR C-18 analytical column (4.8 mm × 150 mm; 5 μm particle size, (Merck, Darmstadt, Germany)) controlled at 45 °C was used as the stationary phase, with a gradient elution program of a 30 min run-time. Mobile phase compositions and flow rate were developed as shown in Supplementary Material S2. The injection volume was 10 μL and the UV absorption was measured at 280 and 425 nm using a diode array detector [28,29,30]. The method validation parameters consisted of linearity, the limit of detection (LOD) and limit of quantification (LOQ), accuracy, and precision [31,32]. Details on the preparation of standard and internal standard solutions are provided in Supplementary Material S3.

### 2.7. Photostability Evaluation

#### 2.7.1. Chemical Stability of Active Markers in the Extract Mixtures

The photostability of extracts was tested following the Q1B ICH guideline regarding Photostability Testing of New Drug Substances and Products [23,33]. To compare the stabilization ability of added antioxidants, single extracts and their combinations by weight ratios such as turmeric extract (TE), chili extract (CE), a 1:1 turmeric–chili mixture (TCE), a 1:1:1 turmeric–chili mixture with coffee extracts (TCE-C), a 1:1:1 turmeric–chili mixture with vitamin E (TCE-E), and a 1:1:1 turmeric–chili mixture with avocado oil (TCE-A) were prepared and irradiated with UV and white light for the total of 72,000–84,000 lux hours [34]. At predetermined time intervals (0, 4, 8 and 12 h), the UV light- and white light (6000–7000 lux)-irradiated samples were adjusted to the initial volume, pipetted, and mixed with the IS (1:1 ratio) in amber HPLC vials before the analysis with HPLC. The concentrations of curcumin and capsaicin were determined and plotted against the time exposed to light. The rate constant (k) was calculated from the resulting slope and used to determine the half-life.

T_1/2_ = 0.693/k (2)

#### 2.7.2. Chemical Stability of Active Markers in Nanoemulsion

The photostability of the extract-loaded nanoemulsion was tested following the ICH guideline as mentioned in the photostability test [34]. One gram of the extract-loaded nanoemulsion was mixed with 5 mL of ethanol. After that, the sample was centrifuged at 10,000 rpm for 10 min. The supernatant was collected and mixed with IS at a ratio of 1:1. The amount of the active compounds in the formulation was analyzed by HPLC and the remaining substances were quantified and calculated for their half-lives.

#### 2.7.3. Antioxidative Stability of the Extract Mixture

For the determination of their ability to protect against oxidation reactions upon UV and white light irradiation, the antioxidative activity of the extracts fortified with antioxidants was determined before and after light exposure (8 h) using the 2,2-diphenyl-1-picrylhydrazyl (DPPH) and the ferric reducing antioxidant power (FRAP) assays. The percent of the remaining antioxidant activity was shown for functional stability protection.

##### DPPH Assay

The scavenging activity of extracts on DPPH radicals was investigated following a previously described method [26]. Mixing 180 μL of DPPH solution in ethanol with 20 μL of each sample in a 96-well plate, followed by incubation for 30 min in the dark, was used as the protocol for the analysis. A multimode detector (Beckman Coulter^®^ GmbH, Vienna, Austria) was used for the detection of DPPH activity at 517 nm. The percentages of the remaining DPPH values of the extract solutions were calculated from the baseline and after light exposure values.

##### FRAP Assay

The ferric reducing antioxidant power of the extract mixture was detected by following a previously described method [26]. The FRAP reagent included 0.3 M acetate buffer (pH 3), 10 mM TPTZ in 40 mM of 37% *v*/*v* HCl solution, and 20 mM ferric chloride solution at a ratio of 10:1:1. After mixing 180 μL of FRAP reagent with 20 μL of each sample in a 96-well plate, the mixture was incubated at room temperature for 5 min and then measured at 595 nm by a multimode detector. The absorbance was analyzed using a ferrous sulfate (FeSO_4_) standard curve with the following equation:

y = 0.1405x + 0.0287(3)

where y is the absorbance at 595 nm and x is the concentration of FeSO_4_ (r^2^ = 0.9926).

The FRAP value was reported as the power of the ferric reducing antioxidant by the following equation:

FRAP value ((mM FeSO_4_)/g) = ([(A − B) − 0.0287])/0.1405 (4)

where A is the absorbance of the sample, and B is the absorbance of the blank solution.

The percentages of the remaining FRAP values of the extract solutions were calculated from the baseline and after light exposure values.

### 2.8. Density Functional Theory (DFT) Calculation

To investigate the structure and antioxidant properties of compounds in the gas phase, Density Functional Theory (DFT) calculations were carried out at the Becke, 3-parameter, Lee–Yang–Parr (B3LYP) level [35,36] with the 6-31G (d, p) basis set [37].

#### 2.8.1. Interaction Energy (E_int_)

To determine whether the compounds in coffee (caffeine or chlorogenic acid) can stably interact with curcumin or capsaicin, and whether the two active ingredients from turmeric and chili can interact when put together, the interaction energies between each of the two molecules—caffeine–curcumin, caffeine–capsaicin, chlorogenic acid–curcumin, chlorogenic acid–capsaicin, and curcumin–capsaicin—were calculated according to the following formula:

E_int_ = E_dimer A_B_ – (E_monomerA_ + E_monomerB_) (5)

where E_dimer A_B_ is the optimized energy of the dimer between compounds A and B, E_monomerA_ is the optimized energy of molecule A, and E_monomerB_ is the optimized energy of molecule B. A negative E_int_ denotes an exothermic process and a positive E_int_ denotes an endothermic process [38].

#### 2.8.2. HOMO-LUMO Energy Gap (HLG) Analysis

The frontier molecular orbital (FMO), including the highest occupied MO (HOMO) and the lowest unoccupied MO (LUMO), offers information about a chemical structure’s kinetic stability, chemical reactivity, biological characteristics, conductivity, and other attributes. The HLG values are considered to explore the electronic properties and reactivity of the considered complexes. This quantity’s operational equation is as follows:

HLG = E_LUMO_ − E_HOMO_ (6)

where E_HOMO_ and E_LUMO_ are the HOMO and the LUMO energies, obtained from DFT calculation, respectively. A high HLG value typically indicates greater molecular stability and lower chemical reactivity. The antioxidant potential of a compound can be inferred from its HOMO energy level; lower HOMO values suggest weaker electron-donating ability and, consequently, diminished antioxidant capacity [39,40,41].

#### 2.8.3. Global Reactive Descriptors

Global reactive descriptors by Koopman’s theorem can be calculated from HOMO and LUMO energies by using the equations below:

Ionization potential;   IP = −E_HOMO_ (7)

Electron affinity;   Ea = −E_LUMO_ (8)

(9) $$\mathrm{Electronegativity};\;\;\;\;\chi =\frac{IP+Ea}{2}$$

(10) $$\mathrm{Hardness};\;\;\;\eta =\frac{IP-Ea}{2}$$

(11) $$\mathrm{Softness};\;\;\;S=\frac{1}{2\eta }$$

Chemical potential;    µ = −(χ)(12)

The ionization potential (*IP*) and electron affinity (*Ea*) of a molecule are directly determined by the negative of its HOMO and LUMO energies, respectively. The system’s overall stability is reflected in the global hardness, which essentially denotes the atoms’, ions’, or molecules’ resistance to the deformation or polarization of their electron clouds in the face of minor perturbations that arise during chemical reactions. Chemical softness, which is inversely proportional to chemical hardness, is a molecule’s ability to take electrons and is more directly tied to the groups or atoms that make up that molecule [37,40,41,42].

### 2.9. Statistical Analysis

Data were analyzed using GraphPad Prism version 10.2.0 for Windows (GraphPad Software, Boston, MA, USA). All experiments in this study were performed in triplicate. The results are presented as the mean ± standard deviation (SD) and mean ± standard error of the mean (SEM). Statistical significance was assessed using one-way ANOVA followed by Dunnett’s multiple comparisons test at a probability level of 0.05.

## 3. Results and Discussion

### 3.1. RP-HPLC Method Validation for Determination of Active Markers

**Figure 1:**
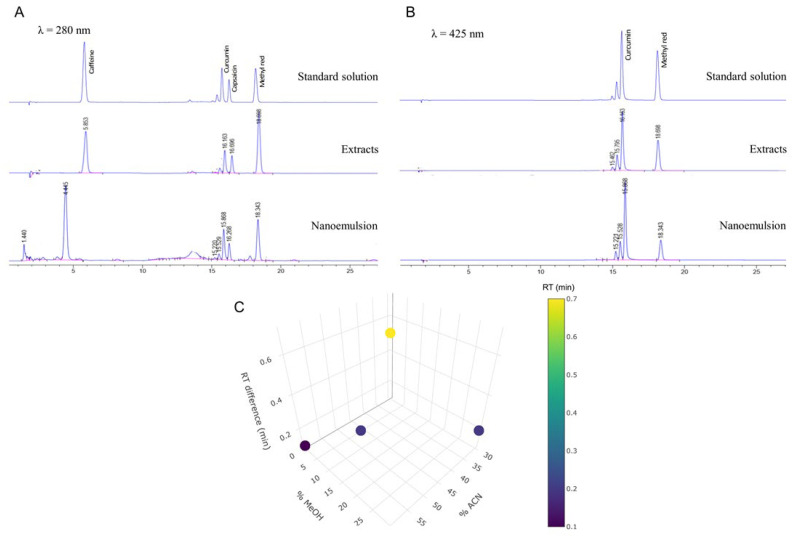
Representative HPLC chromatograms of caffeine, curcumin, capsaicin, and methyl red (75 μg/mL) in standard solution, extracts, and nanoemulsion observed at 280 nm (**A**) and 450 nm (**B**). Separation of curcumin and capsaicin peaks represented as retention time difference at various ratios of acetonitrile and methanol (**C**); color bar indicates retention time difference (min).

For stability monitoring of the active markers in the combined samples (curcumin, capsaicin, and caffeine), an RP-HPLC method was developed to simultaneously determine all three compounds in a single run. The method utilized gradient elution with optimized solvent composition and a column temperature of 45 °C to achieve effective separation, despite the nearly logP values (curcumin, logP 3.2, and capsaicin, logP 3.6) [43,44]. However, caffeine, which is a more polar molecule (logP = 0.85), required a weaker solvent in the separation [45]. A photodiode array detector enabled dual-wavelength detection at 280 nm (for caffeine and capsaicin) and 425 nm (for curcuminoids) [46,47,48]. Representative chromatograms, including a sample containing 100 µg/mL caffeine, are shown in Figure 1. The results of the accuracy and precision evaluation are summarized in Supplementary Material S4. Acceptable characteristics of the analytical method were proven for the analysis of curcumin, capsaicin, and caffeine in the extracts, formulations, and stability studies.

### 3.2. Nanoemulsion Formulation

#### 3.2.1. Solubility Study of the Extracts in Various Vehicles

Turmeric, chili, and coffee extracts were evaluated for their solubility in various vehicles according to the criteria in British Pharmacopoeia 2019 [24]. The results are presented in Supplementary Material S5. Arabica roasted coffee bean extract was freely soluble in avocado oil, DMSO, methanol, and PEG 400 and soluble in ethanol and 10% Tween 80. Turmeric extract was freely soluble in avocado oil, DMSO, and PEG 400 and soluble in 10% Tween 80. In contrast, chili extract can dissolve in DMSO and methanol. Based on these findings, 10% Tween 80, PEG 400, and avocado oil were selected as suitable solubilizers for the extracts and used as the internal phase of the nanoemulsions.

#### 3.2.2. Preparation of Extract-Loaded Nanoemulsions

Polyacrylamide (and) C13-14 Isoparaffin (and) Laureth-7 (HLB 14) and polyglyceryl-3 rice branate (HLB 11) as natural emulsifiers varied from 1 to 6% *w*/*w* in the pre-emulsion formation step [49,50]. In contrast to other studies, the formulations containing either polyglyceryl-3 rice branate or Polyacrylamide (and) C13-14 Isoparaffin (and) Laureth-7 at 3–4% after homogenizing using a high-shear homogenizer exhibited homogeneous and stable emulsions without phase separation after 24 h at room temperature. The optimal emulsifier concentrations for stabilizing oil-in-water emulsions under the tested conditions were obtained, as detailed in Supplementary Material S6. To further produce nanoemulsions, the selected pre-emulsion formulation containing polyglyceryl-3 rice branate at 4% was subjected to a high-shear homogenization process, followed by high-pressure homogenization at 500 bar for 5, 10, or 15 min. The results indicated that increasing the homogenizing time significantly reduced both particle size and the polydispersity index (PDI) value (*p* < 0.05). The narrowest PDIs (0.12 ± 0.02), the smallest particle sizes (179.52 ± 2.43 nm), and low zeta potential (−46.38 ± 2.48 mV) were observed in the nanoemulsion obtained after 15 min of homogenization, as shown in Table 3. The collective size reduction processes yielded the desirable nanoemulsion characteristics for transdermal delivery. The energy required to form the submicron emulsions is one of the key characteristics of the nanoemulsion compared to the transparent microemulsion [51]. After passing the pre-emulsions through high-pressure homogenization, large droplets of pre-emulsions were broken down into nano-sized droplets. Previous studies highlighted that the number of passages affects the particle size of nanoemulsions, typically requiring several repetitions until a constant droplet size is achieved [25,52,53]. Nanoemulsions with droplet sizes typically ranging from 20 to 200 nm are suitable for dermal applications, and these sizes can be achieved depending on the composition and preparation methods. The PDI, ranging from 0 to 1, reflects the uniformity of droplets in the formulation. A PDI lower than 0.2 is considered a desirable value, indicating good particle size distribution [54,55]. Zeta potential, describing the surface charge on droplets, is crucial for stability, generating repulsion forces between droplets to prevent particle agglomeration. A desirable zeta potential is at least ±30 mV [54,56,57]. All the criteria were met by formulation 4 (F4), the one containing 4% polyglyceryl-3 rice branate, which showed both stability and structural integrity, making it suitable for encapsulating active compounds, as detailed in Supplementary Material S7.

A high-pressure homogenizer with a run time of 15 min was suitable for producing the formulated pre-emulsion and extract-loaded nanoemulsions. To entrap the bioactive compounds in the formulation, capsaicin—a bioactive constituent of chili extract with potent anti-inflammatory and antioxidant properties—was included at a fixed concentration of 0.05% due to its pungent odor and irritant potential [58]. Meanwhile, the concentrations of curcumin and caffeine were optimized and evaluated for their combined effects. In this study, 10% Tween 80 was used to enhance the solubilization of the extract, thereby facilitating the incorporation of active extracts into the nanoemulsions. The formulation containing curcumin/capsaicin/caffeine at a 3:2:3 ratio (TCE-C-NE-3) exhibited immediate phase separation. Similarly, the 2:2:2 ratio (TCE-C-NE-2) showed signs of instability following the stability test. In contrast, the 1:2:1 formulation (TCE-C-NE-1) demonstrated favorable physical appearance, pH, and color and remained stable when stored at 4 °C, as shown in Table 4. However, storage at 30 °C resulted in a significant increase in droplet sizes, which may be attributed to Ostwald ripening or coalescence, suggesting the nanoemulsions should be stored at 4 °C or immediately processed to form finished products. Similar destabilization was observed by Nazarzadeh et al. in their study on the physical stability of nanoemulsions under elevated temperature conditions [59]. A nanoemulsion system is considered physically stable if there are only small changes in the droplet size and PDI after a certain storage period.

#### 3.2.3. Entrapment Efficiency of Extract-Loaded Nanoemulsions

**Figure 2:**
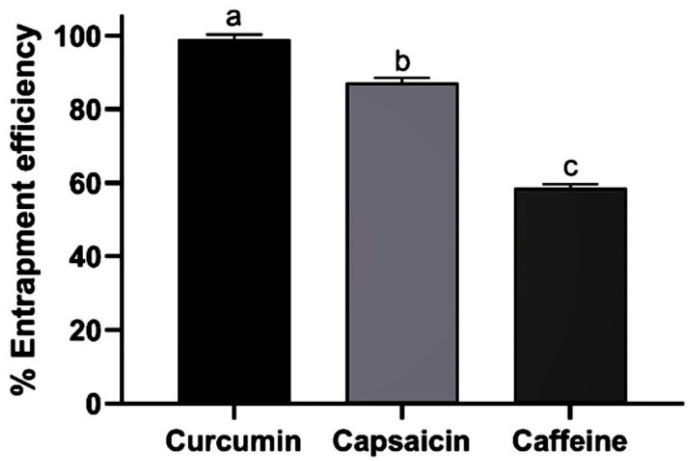
Entrapment efficiencies of curcumin, capsaicin, and caffeine. Values expressed as mean ± SEM (n = 3); a, b, c show significant differences between samples at *p* < 0.05.

The 1:2:1 formulation (TCE-C-NE-1) was further subjected to entrapment efficiency (%EE) analysis. The entrapment efficiencies of curcumin, capsaicin, and caffeine in the extract-loaded nanoemulsion were found to be 99.2 ± 2.20%, 87.2 ± 2.20%, and 58.55 ± 1.88%, respectively, as shown in Figure 2. The optimal ratio of lipid, surfactant, and aqueous phases in the nanoemulsion, along with the optimized incorporation of extracts into the appropriate phase, significantly affects the entrapment efficiency. The polarity of the extracts is a key factor in determining whether they should be incorporated into the aqueous or lipid phase [60]. Caffeine, being hydrophilic, with a logP value of 0.85, exhibits low entrapment efficiency due to its better solubility in the water phase [61,62]. In contrast, curcumin and capsaicin are hydrophobic molecules with logP values of 3.2 and 3.6, respectively [43,44,45]. Therefore, curcumin and capsaicin can be entrapped in the oil phase of the formulation and reveal higher entrapment efficiencies.

### 3.3. Effects of Antioxidants on Photostability of Curcumin and Capsaicin in the Extract Mixture

#### 3.3.1. Photostability on Turmeric and Chili Extract Solutions and Nanoemulsions

**Figure 3:**
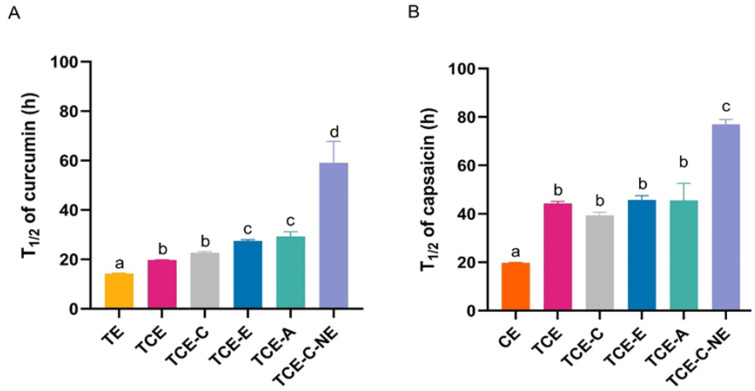
The half–lives of curcumin (**A**) and capsaicin (**B**) in turmeric extract (TE), chili extract (CE), turmeric–chili mixture (TCE), turmeric–chili–coffee mixture (TCE-C), turmeric–chili mixture with vitamin E (TCE-E), turmeric–chili mixture with avocado oil (TCE-A) and turmeric–chili–coffee mixture in nanoemulsions (TCE-C-NE). Values are expressed as mean ± SD (n = 3). Different letters (a, b, c, d) indicate significant differences between samples at *p* < 0.05.

Health products containing natural substances are highly promoted; this is particularly the case for turmeric and chili extracts due to their antioxidant properties. However, the major active constituents—curcumin and capsaicin—are unstable when exposed to light, leading to degradation. This degradation can be analyzed through the degradation rate constant (k) and half-life (T_1/2_). Curcumin degrades more rapidly (k = 490 × 10^−3^ ± 0.00 h^−1^, T_1/2_ = 14.20 ± 0.31 h) compared to capsaicin (T_1/2_ = 19.71 ± 0.33 h), as shown in Figure 3A,B, while the degradation rate constant (k) is provided in Supplementary Material S8. In an aqueous environment, the phenoxyl radicals of curcumin decayed within a few hundred microseconds. This instability can be accelerated by light or oxidative reactions. Bicyclopentadione has been identified as the major compound formed during the autoxidation of curcumin [63]. In the present study, capsaicin exhibited greater photostability than curcumin, which may be attributed to its more stable molecular structure and lower susceptibility to autoxidation. The addition of coffee extract enhanced the stability of both curcumin and capsaicin, increasing their half-lives to 22.64 ± 0.84 h and 39.38 ± 2.18 h, respectively. Furthermore, the inclusion of vitamin E and avocado oil in the curcumin and capsaicin mixtures demonstrated an additional stabilizing effect. Accordingly, nanoemulsions containing turmeric and chili extracts were developed and formulated with coffee extract, vitamin E, and avocado oil as stabilizing excipients. The photo-induced degradation study revealed significantly decreased degradation rate constants of curcumin and capsaicin in the nanoemulsions, at 120 × 10^−3^ ± 0.00 h^−1^ and 90 × 10^−3^ ± 0.00 h^−1^, respectively, as shown in Supplementary Material S8. The corresponding half-lives were 59.08 ± 3.89 h for curcumin and 76.94 ± 3.62 h for capsaicin, representing increases of 416% and 390%, respectively, compared to the extracts alone (Figure 3A,B). The improved stability of the active compounds in the nanoemulsions can also be attributed to the entrapment within the internal phase, which provided protection against UV and visible light exposure.

The stabilization of curcumin and capsaicin in nanostructured lipid carriers (NLCs) has previously been reported, revealing that an appropriate ratio of curcumin, capsaicin, and chlorogenic acid significantly enhanced their photostability, with the half-life of curcumin increasing from 9.63 h to 57.75 h and that of capsaicin from 6.79 h to 38.50 h [20]. These findings are consistent with the results observed in the present study. In the present investigation, the photostability of curcumin and capsaicin in the presence of coffee extract was markedly improved, with the half-life of curcumin increasing from 14 h to nearly 60 h and that of capsaicin from approximately 20 h to 80 h. This pronounced stabilization effect could be attributed to the interruption of chain reactions involving curcumin free radicals, which are highly susceptible to autoxidation, by the antioxidants present in the coffee extract, vitamin E, and natural oils in the nanoemulsions. The degradation process was effectively delayed by incorporating various redox-active antioxidants, such as gallic acid, ascorbate, vitamin C, TBHQ, caffeic acid, rosmarinic acid, and Trolox, all of which significantly enhance the stability of curcumin by up to ~300% compared to curcumin without other antioxidants [64]. Moreover, the stabilization could be partly attributed to the intermolecular interactions, potentially involving hydrogen bonding, electrostatic forces, or van der Waals interactions [65]. Capsaicin is also prone to degradation, but the available data are limited. Published studies suggest that encapsulation is the most widely adopted technique for improving the stability of capsaicin against oxygen, light, pH, and humidity [66].

#### 3.3.2. Photostabilizing Efficiency of Natural Stabilizers

**Figure 4:**
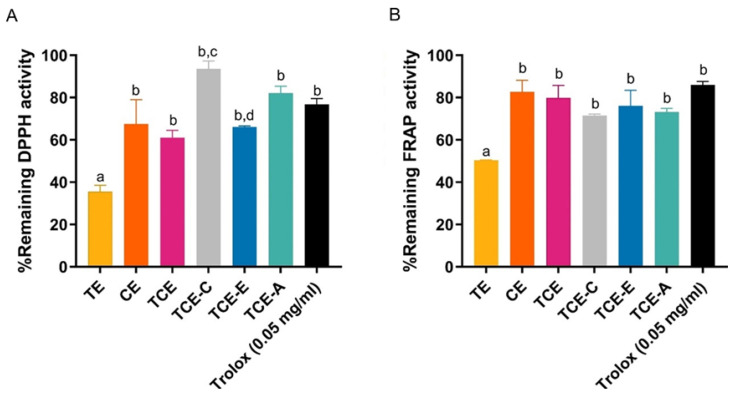
The remaining percentages of antioxidant activity measured (**A**) by DPPH assay and (**B**) FRAP assay after 12 h exposure to UV and white light for turmeric extract (TE), chili extract (CE), turmeric–chili mixture (TCE), turmeric–chili–coffee mixture (TCE-C), turmeric–chili mixture with vitamin E (TCE-E), turmeric–chili mixture with avocado oil (TCE-A), and Trolox (50 μg/mL). Values are expressed as mean ± SD (n = 3). Different letters (a, b, c, d) indicate significant differences between samples at *p* < 0.05.

This section highlights the comparative effectiveness of natural antioxidants (coffee extract, vitamin E, avocado oil) as stabilizers for the antioxidant activity of the extract mixture after UV and white light exposure based on the remaining percentages of antioxidant activity determined by DPPH and FRAP assays. The results show that the highest DPPH free radical scavenging remaining activity of 93.63 ± 2.86% was observed for the addition of coffee extract to the turmeric–capsaicin mixture (TCE-C) (Figure 4A). The results of the FRAP assay exhibited comparable remaining antioxidant activity (>70% remaining) in all samples, except for the turmeric solution (~50%) (Figure 4B). Combining different antioxidants not only resulted in a synergistic effect, where the total antioxidant activity was greater than the sum of individual activities [67], but it also generated degradation protection against photo- or UV activation. A decrease in the half-life of a compound correlates with a reduction in antioxidant activity, as was also reported by Lee et al., who showed that curcumin degrades more rapidly when exposed to UV radiation for 24 h, leading to a significant reduction in DPPH activity—from around 60% to 50% inhibition [68]. The stability of these active antioxidants ensures their prolonged efficacy, making them particularly valuable in formulations intended for chronic administration, where consistent performance and sustained activity are essential.

### 3.4. Quantification of Active Compounds in Crude Coffee Extract

**Figure 5:**
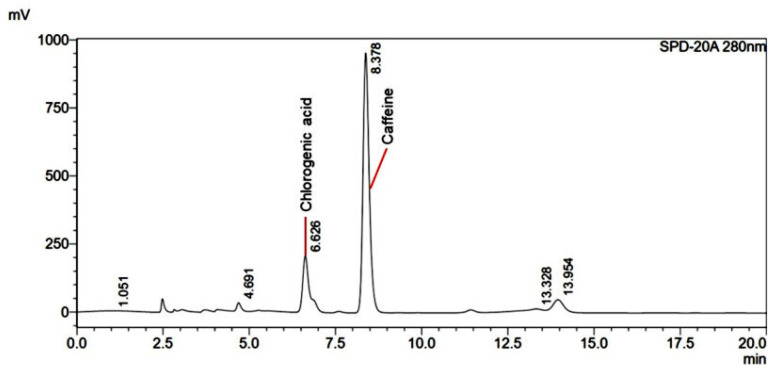
HPLC chromatograms of coffee extracts using HPLC at a wavelength of 280 nm.

In the evaluation of remaining activity via DPPH and FRAP assays, coffee extract showed significant protective activity against photodegradation. A question arose about which active compound in coffee extract is responsible for stabilization. The active compounds in the coffee extract were analyzed using HPLC-UV. The data obtained from the experiment showed that the major components in the coffee extract were caffeine and chlorogenic acid, with retention times of 6.62 and 8.37 min, respectively. All extracts showed major peaks at the same standard retention time (Figure 5). The data obtained from the experiment showed that the percentage of caffeine and chlorogenic acid in the coffee extract was 6.62 ± 0.11 and 3.11 ± 0.35, respectively. Therefore, these two compounds were evaluated for the protective activity against photodegradation in comparison with the crude extracts.

### 3.5. Photostabilizing Efficiency of Coffee Extracts and Their Isolated Components

**Figure 6:**
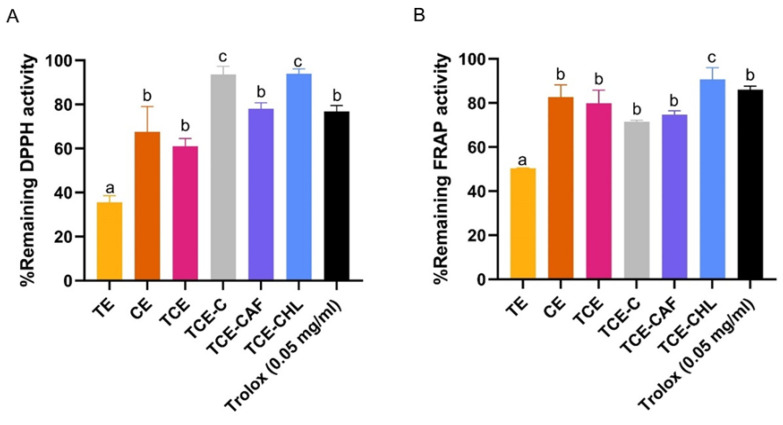
The remaining percentages of antioxidant activity measured (**A**) by DPPH assay and (**B**) FRAP assay after 12 h exposure to UV and white light on turmeric extract (TE), chili extract (CE), turmeric–chili mixture (TCE), turmeric–chili–coffee mixture (TCE-C), turmeric–chili–caffeine mixture (TCE-CAF), and turmeric–chili–chlorogenic acid mixture (TCE-CHL). Values are expressed as mean ± SD (n = 3). Different letters (a, b, c) indicate significant differences among samples at *p* < 0.05.

The identification of the major constituents of the coffee extracts confirmed the presence of chlorogenic acid and caffeine as the predominant compounds. To evaluate their comparative photoprotective efficiencies, these isolated constituents were assessed alongside the crude extract and Trolox using DPPH and FRAP assays, measuring the retention of antioxidant activity following 12 h of exposure to UV and white light. At equivalent concentrations, chlorogenic acid exhibited a markedly higher photostabilizing efficiency than caffeine in both assays. Notably, the residual antioxidant activity of the chlorogenic acid-treated extract (TCE-CHL) surpassed that of Trolox, a widely recognized reference antioxidant (Figure 6A,B). The potent antioxidative properties of chlorogenic acid can be attributed to its phenolic hydroxyl groups, which effectively scavenge free radicals. This compound is abundant in green coffee beans and is increasingly recognized for its substantial antioxidant and anti-inflammatory activities [69]. In contrast, caffeine demonstrated comparatively weaker and less consistent antioxidant performance under the same conditions [69].

### 3.6. Chemical and Structural Features Influencing the Stability of Curcumin and Capsaicin

#### 3.6.1. Interaction Energy (E_int_)

The stability of curcumin and capsaicin in the photostability test was confirmed by their interaction energies (E_int_). Molecular recognition and binding depend on interaction energies. Complementing the experimental findings, DFT calculations using the B3LYP/6-31G (d, p) method were employed to investigate the interactions between various pairs of bioactive molecules. The interaction energies between caffeine–capsaicin, chlorogenic acid–capsaicin, curcumin–capsaicin, chlorogenic acid–curcumin, and caffeine–curcumin were −11.0191, −7.7435, −7.6807, −5.4593, and −2.2088 kcal/mol, respectively, as shown in Table 5. This result shows that every system is an exothermic process. This indicates that all five pairs of substances can interact and form stable complexes. Caffeine and capsaicin were the pair of substances that could most easily interact and were the most stable because their interaction energy value was the most negative. This was followed by the interaction between chlorogenic acid and capsaicin, and that between curcumin and capsaicin. Stable complexes and strong binding are related to the ability to diminish or avoid the contact of the active compound with free radicals from photoreaction; they can enhance the photostability of the compound. This means that the compound does not quickly degrade when exposed to light, and therefore the efficiency of half-lives can be longer [70].

#### 3.6.2. HOMO-LUMO Energy Gap (HLG) Analysis and Global Reactive Descriptors

Frontier orbitals offer insights into the highest occupied molecular orbitals (HOMOs) and the lowest unoccupied molecular orbitals (LUMOs), which represent the capability of molecules to donate or accept electrons. Molecules with higher HOMO levels tend to be better electron donors, while molecules with lower LUMO have the capability to accept electrons and effectively neutralize free radicals. These parameters are crucial for describing antioxidant ability due to their role in electron transfer [71].

**Figure 7:**
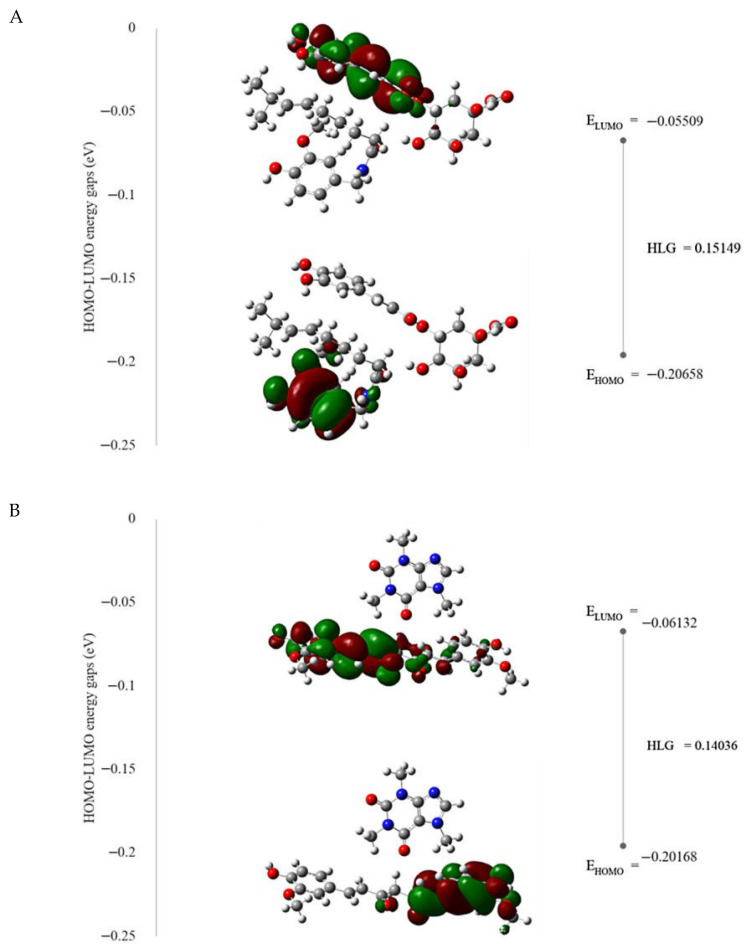
HOMO-LUMO energy gaps of chlorogenic acid–capsaicin (**A**) and caffeine–curcumin (**B**) at 6-31G (d, p) level.

The results of HOMO, LUMO, and HLG of all five single substances and five pairs of substances, together with the previous interaction energy values, showed that when curcumin and capsaicin, two active substances, come together, the HLG value is very narrow (HLG), indicating better antioxidant activity but also instability. The narrow HLG of the curcumin–capsaicin complex is associated with enhanced antioxidant activity because these molecules can readily donate and accept electrons, which is a key mechanism of neutralizing free radicals [71]. While reactivity is increased, the complex also becomes less stable due to its susceptibility to degradation, especially under environmental stress such as light exposure. This instability is consistent with earlier experimental results, which showed that the curcumin–capsaicin mixture is less stable. However, the presence of chlorogenic acid tends to bind and stabilize capsaicin because it has the widest HLG value compared to other pairs of substances (Table 6). The high HLG of the chlorogenic acid–capsaicin complex indicates greater resistance to chemical degradation, which can extend its half-life [71]. While caffeine and capsaicin interact easily, they are the second most stable. For the pair of chlorogenic acid and capsaicin, HOMO appears all over the aromatic ring and the amide region of capsaicin, and LUMO develops over the caffeic acid and ester bond of chlorogenic acid (Figure 7A).

In addition, there is an interesting observation for the HLG value: the caffeine in coffee interacts with curcumin and causes the HLG value to be wider than the HLG value of the single molecule curcumin. This interaction preserves the HOMO-LUMO distribution of curcumin and supports its antioxidant activity, as the key hydrogen atom transfer (HAT) mechanism remains intact (Figure 7B) [72]. This suggests that caffeine may help stabilize curcumin. HOMO and LUMO remain localized on the structure of curcumin.

The global reactive descriptors calculated from HOMO and LUMO are displayed in Table 7. The results show that the curcumin–capsaicin complex is more reactive than curcumin or capsaicin as single molecules. The chemical hardness and softness values of chlorogenic acid–capsaicin are calculated at 0.07575 and 6.60110 eV, respectively. The chlorogenic acid and capsaicin pair is harder and more stable than other substances. This is related to the study of Boulmokh et al., in which the analysis of the highest HOMO-LUMO energies indicated enhanced reactivity, supporting the observed antioxidant activity and molecular stability [72].

#### 3.6.3. The Proposed Interaction of Compounds in Roasted Arabica Coffee Bean Extract with Capsaicin and Curcumin

**Figure 8:**
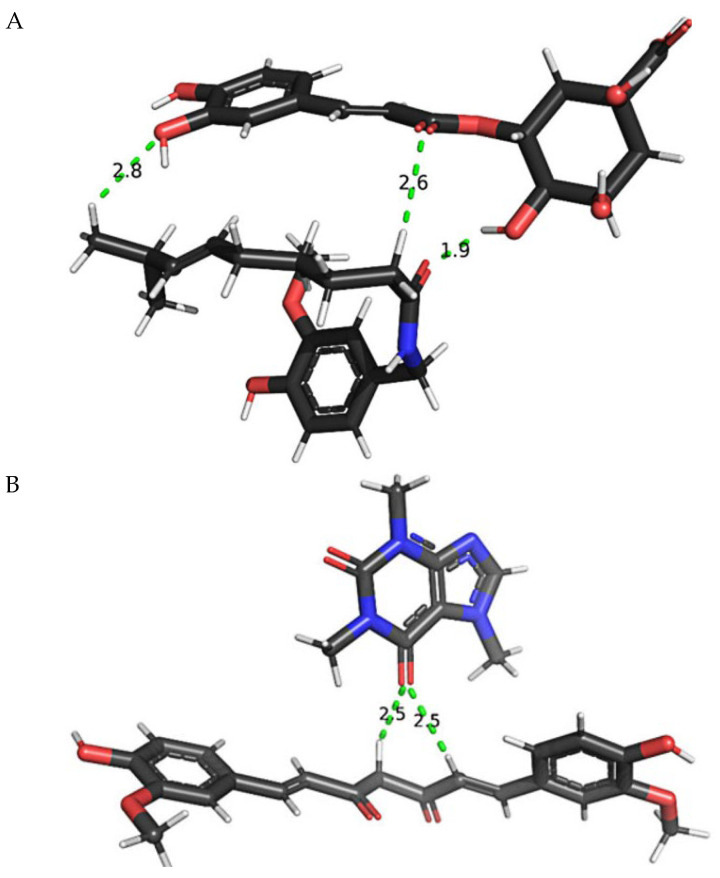
Alignment between capsaicin and chlorogenic acid (**A**) and alignment between curcumin and caffeine (**B**) after DFT B3LYP/6-31G (d, p) calculation.

From HLG analysis, the compounds in coffee extract that are likely to bind and stabilize capsaicin and curcumin are chlorogenic acid and caffeine, respectively. Based on measuring the distance between atoms with the PyMOL2 program [73], it is expected that capsaicin and chlorogenic acid form hydrogen bonds [74]. It may occur in three bonds, as shown in Figure 8A. Meanwhile, caffeine also forms a hydrogen bond with curcumin. It occurs between the oxygen carbonyl of caffeine and two hydrogens of curcumin (Figure 8B).

In summary, this study provides valuable insight into the antioxidant activity and stability of curcumin and capsaicin using experimental and computational approaches. The findings suggest that the presence of active compounds in coffee extract, such as caffeine and chlorogenic acid, can significantly enhance the stability and reactivity of capsaicin and curcumin. This offers potential applications in developing novel formulations that have synergistic effects that enhance the bioactivity of these compounds.

Based on the interaction energy analysis, which indicates the binding affinity of active compounds, it was found that caffeine has the highest affinity for binding with capsaicin. This result leads to greater stabilization of capsaicin, resulting in the capsaicin–caffeine complex being the most stable, as indicated by the increased half-life of capsaicin in the TCE-C mixture. Chlorogenic acid also helps to stabilize capsaicin, though to a lesser extent. However, chlorogenic acid can bind well with curcumin, thereby protecting curcumin from degradation, as evidenced by the increased half-life of curcumin. Therefore, the addition of coffee seed extract, which contains caffeine and chlorogenic acid, helps to increase the stabilization of the turmeric–chili mixture, especially capsaicin. Regarding antioxidant capacity, curcumin demonstrated the highest reactivity but was also the least stable, as indicated by its smaller HLG (HOMO-LUMO Gap). Conversely, capsaicin exhibited lower reactivity but greater stability, as indicated by its wider HLG. However, when turmeric and chili extracts are combined, the curcumin–capsaicin pair becomes less reactive compared to curcumin alone, preserving more antioxidant activity in the mixture than in the individual substances, as shown in the % remaining DPPH and FRAP activity. The addition of coffee extract further stabilized the complex, particularly through the chlorogenic acid–capsaicin interaction, as indicated by the increased HLG, consistent with the increased % remaining DPPH and FRAP activity in the mixture. From the binding characteristics of chlorogenic acid with capsaicin, it is evident that chlorogenic acid binds with capsaicin in a closed form, protecting the amide linkage from degradation, and the vanillyl moiety of capsaicin and the catechol group of chlorogenic acid still have space to bind free radicals. Similarly, caffeine can bind with curcumin, leading to a more stable complex and thereby preserving more antioxidant activity in the mixture than in the individual substances, as evidenced by the increased percentage of remaining DPPH in the mixture. By binding to the carbon linker chain, caffeine helps curcumin retain its antioxidant activity by keeping the o-methoxy phenolic group available to bind with free radicals. Therefore, caffeine and chlorogenic acid, in addition to their antioxidant properties, play a crucial role in preserving the antioxidant capacity of curcumin and capsaicin within the mixture.

## 4. Conclusions

This study demonstrates that incorporating natural antioxidants—particularly roasted *Coffea arabica* extract and vitamin E-rich oil—into nanoemulsion formulations significantly enhances the photostability and antioxidant performance of curcumin and capsaicin in aqueous environments. Improved stability was evidenced by increased half-lives and enhanced antioxidation capacity, as determined through quantitative analysis and DPPH/FRAP assays. These findings originally highlight the promising role of coffee-derived polyphenols in stabilizing sensitive bioactives from turmeric and chili extracts, offering a natural strategy to augment the efficacy and self-life of plant-based health and cosmetic products. Beyond formulation stability, this work provides valuable insight into the molecular interactions governing antioxidant protection within complex delivery systems. The proposed molecular recognition and binding are based on interaction energies between curcumin and capsaicin and major compounds in coffee bean extract, reported here for the first time. Future investigations should focus on improving the stability of the final products and expanding their translational potential in pharmaceutical and nutraceutical applications.

## Figures and Tables

**Table 1:** Formulation components of the pre-emulsion systems (% *w*/*w*).

Ingredients	F1-12
1. Oil mixture	21
2. Emulsifier	1–6
3. Polyethylene glycol 400	5
4. Phenoxyethanol	1
5. Water	q.s.100

**Table 2:** Formulations of the extract component load in the nanoemulsion system (% *w*/*w*).

Ingredients	TCE-C-NE-1	TCE-C-NE-2	TCE-C-NE-3
1. Turmeric extract	0.025	0.050	0.075
2. Chili extract	0.050	0.050	0.050
3. Coffee extract	0.025	0.050	0.075
4. Oil mixture	21	21	21
5. Emulsifier (selected)	4	4	4
6. Polyethylene glycol 400	5	5	5
7. Phenoxyethanol	1	1	1
8. Tween 80	10	10	10
9. Water	q.s.100	q.s.100	q.s.100

Note: TCE-C-NE-1, TCE-C-NE-2, and TCE-C-NE-3 represent nanoemulsion formulations containing turmeric, chili, and coffee extracts at increasing concentrations with fixed ratios of 1:2:1, 2:2:2, and 3:2:3, respectively.

**Table 3:** Mean particle size, PDI, and zeta potential of the selected unloaded nanoemulsions (F4), which were prepared using a high-pressure homogenizer for different amounts of time. Different letters (^a^,^b^,^c^) indicate significant differences between samples at *p* < 0.05.

Record	Unloaded Nanoemulsions		
Homogenization Time (min)	5	10	15
Average particle size (nm)	303.12 ± 1.83 ^a^	251.74 ± 1.9 ^b^	179.52 ± 2.43 ^c^
Average PDI	0.35 ± 0.01 ^a^	0.32 ± 0.05 ^a^	0.12 ± 0.02 ^b^
Average zeta potential (mV)	−48.21 ± 0.46 ^a^	−45.35 ± 2.83 ^a^	−46.38 ± 2.48 ^a^

**Table 4:** Particle sizes, PDI, and zeta potential of extract-loaded nanoemulsions (TCE-C-NE-1) before and after stability study. Different letters (^a^,^b^) indicate significant differences between samples at *p* < 0.05.

		TCE-C-NE-1			
Parameters	Day 0	30 °C		4 °C	
		30 Days	60 Days	30 Days	60 Days
Particle size (nm)	170.2 ± 1.32 ^a^	246.03 ± 0.45 ^b^	248.86 ± 1.96 ^b^	180.1 ± 1.75 ^a^	182.7 ± 1.75 ^a^
PDI	0.11 ± 0.03 ^a^	0.12 ± 0.02 ^a^	0.12 ± 0.02 ^a^	0.14 ± 0.03 ^a^	0.11 ± 0.08 ^a^
Zeta potential (mV)	−39.6 ± 2.25 ^a^	−38.7 ± 0.79 ^a^	−37.76 ± 1.62 ^a^	−39.20 ± 0.55 ^a^	−39.60 ± 1.02 ^a^

**Table 5:** The interaction energy (E_int_) between compounds.

Compounds	E(RB3LYP) (Hartree *)	E(RB3LYP) (kcal/mol)
Caffeine	−680.3906	−426,951.5527
Capsaicin	−982.6340	−616,612.1449
Caffeine–Capsaicin	−1663.0421	−1,043,574.7166
E_int_ of Caffeine–Capsaicin	−0.0176	−11.0191
Chlorogenic acid	−1297.5884	−814,249.0481
Capsaicin	−982.6340	−616,612.1449
Chlorogenic acid–Capsaicin	−2280.2347	−1,430,868.9365
E_int_ of Chlorogenic acid–Capsaicin	−0.0123	−7.7435
Curcumin	−1263.5968	−792,918.9962
Capsaicin	−982.6340	−616,612.1449
Curcumin–Capsaicin	−2246.2430	−1,409,538.8218
E_int_ of Curcumin–Capsaicin	−0.0122	−7.6807
Chlorogenic acid	−1297.5884	−814,249.0481
Curcumin	−1263.5968	−792,918.9962
Chlorogenic acid–Curcumin	−2561.1939	−1,607,173.5036
E_int_ Chlorogenic acid–Curcumin	−0.0087	−5.4593
Caffeine	−680.3906	−426,951.5527
Curcumin	−1263.5968	−792,918.9962
Caffeine–Curcumin	−1943.9909	−1,219,872.7577
E_int_ of Caffeine–Curcumin	−0.0035	−2.2088

* 1 Hartree = 627.5095 kcal/mol (Source: https://gaussian.com/constants/, accessed on 10 January 2025).

**Table 6:** HOMO, LUMO, and HLG.

System	HOMO	LUMO	HLG
Curcumin	−0.20480	−0.07210	0.13270
Capsaicin	−0.20221	0.00413	0.20634
Chlorogenic acid	−0.21483	−0.06417	0.15066
Caffeine	−0.21953	−0.03152	0.18801
Curcumin–Capsaicin	−0.19566	−0.06302	0.13264
Chlorogenic acid–Capsaicin	−0.20658	−0.05509	0.15149
Chlorogenic acid–Curcumin	−0.20999	−0.07817	0.13182
Caffeine–Capsaicin	−0.19328	−0.04704	0.14624
Caffeine–Curcumin	−0.20168	−0.06132	0.14036

**Table 7:** Global reactive descriptors of substances.

System	Ionization Potential	Electron Affinity	Electronegativity	Hardness	Softness	Chemical Potential
Curcumin	0.20480	0.07210	0.13845	0.06635	7.53580	−0.13845
Capsaicin	0.20221	−0.00413	0.09904	0.10317	4.84637	−0.09904
Chlorogenic acid	0.21483	0.06417	0.13950	0.07533	6.63746	−0.13950
Caffeine	0.21953	0.03152	0.12553	0.09401	5.31887	−0.12553
Curcumin–Capsaicin	0.19566	0.06302	0.12934	0.06632	7.53920	−0.12934
Chlorogenic acid–Capsaicin	0.20658	0.05509	0.13084	0.07575	6.60110	−0.13084
Chlorogenic acid–Curcumin	0.20999	0.07817	0.14408	0.06591	7.58610	−0.14408
Caffeine–Capsaicin	0.19328	0.04704	0.12016	0.07312	6.83807	−0.12016
Caffeine–Curcumin	0.20168	0.06132	0.13150	0.07018	7.12454	−0.13150

## Data Availability

The original contributions presented in this study are included in the article/Supplementary Material. Further inquiries can be directed to the corresponding author.

## Supplementary Materials

The following supporting information can be downloaded at https://www.mdpi.com/article/10.3390/pharmaceutics17070926/s1, Supplementary Material S1: Physical appearances of pre-emulsions before passing through high pressure homogenizer. Supplementary Material S2. Gradient elution program of the developed HPLC method. Supplementary Material S3. The method for the preparation of standard stock solutions of curcumin, capsaicin, caffeine, and the internal standard (IS). Supplementary Material S4. Intra-day and inter-day accuracy and precision results for curcumin, capsaicin and caffeine. Results are expressed as mean ± SD. Supplementary Material S5. Solubility of arabica roasted coffee bean extract, turmeric extract, and chili extract in various vehicles. Supplementary Material S6. Compositions of pre-emulsion formation. Supplementary Material S7. Physical appearance, pH, mean particle size, PDI, and zeta potential of the selected unloaded nanoemulsions (F4). Different letters (a, b) indicate significant differences among samples at *p* < 0.05. Supplementary Material S8. The degradation rate constants (k) of curcumin and capsaicin.

## Abbreviations

The following abbreviations are used in this manuscript:

HPLC	High-performance liquid chromatography
FRAP	Ferric reducing antioxidant power
DPPH	2,2-diphenyl-1-picrylhydrazyl
T1/2	Half-lives
Eint	Interaction energy
HLG	HOMO-LUMO energy gap
NLC	Nanostructure lipid carriers
DFT	Density functional theory
