# Supplementary material for: Triple-Loaded Nanoemulsions Incorporating Coffee Extract for the Photoprotection of Curcumin and Capsaicin: Experimental and Computational Evaluation
Source: Pharmaceutics. 2025 Jul 17;17(7):926. doi: 10.3390/pharmaceutics17070926 (PMC12299237; doi:10.3390/pharmaceutics17070926)
Supplement: Supplementary file 1 [file pharmaceutics-17-00926-s001.zip › pharmaceutics-3695508-supplementary.pdf]

Supplementary data

Article

Triple-Loaded Nanoemulsions Incorporating Coffee Extract for the Photoprotection of Curcumin and Capsaicin: Experimental and Computational Evaluation

Supplementary Material S1: Physical appearances of pre-emulsions before passing through high pressure homogenizer

Formulations	Concentration of emulsifier (%w/w)	Phase separation	Viscosity	Color
F1	1% Polyglyceryl-3 rice branate	Separate	-	-
F2	2% Polyglyceryl-3 rice branate	Separate	-	-
F3	3% Polyglyceryl-3 rice branate	Not separate	+	Opaque white
F4	4% Polyglyceryl-3 rice branate	Not separate	++	Opaque white
F5	5% Polyglyceryl-3 rice branate	Not separate	+++	Opaque white
F6	6% Polyglyceryl-3 rice branate	Not separate	++++	Opaque white
F7	1% Polyacrylamide (and) C13-C14 Isoparaffin (and) Laureth-7	Separate	-	-
F8	2% Polyacrylamide (and) C13-14 Isoparaffin (and) Laureth-7	Separate	-	-
F9	3% Polyacrylamide (and) C13-14 Isoparaffin (and) Laureth-7	Not separate	+	Opaque white
F10	4% Polyacrylamide (and) C13-14 Isoparaffin (and) Laureth-7	Not separate	++	Opaque white
F11	5% Polyacrylamide (and) C13-14 Isoparaffin (and) Laureth-7	Not separate	+++	Opaque white
F12	6% Polyacrylamide (and) C13-14 Isoparaffin (and) Laureth-7	Not separate	++++	Opaque white

The symbols represent relative levels as follows —+: Low, ++: Medium, +++: High, ++++: Very High.

Supplementary Material S2. Gradient elution program of the developed HPLC method.

Time (min)	Flow rate (mL/min)	1% acetic acid in water	Acetonitrile	Methanol
0	1	83.50	8.25	8.25
7	1	83.50	8.25	8.25
12	1	40.00	55.00	5.00
27	1	40.00	55.00	5.00
28	1	83.50	8.25	8.25
30	1	83.50	8.25	8.25

Supplementary Material S3. The method for the preparation of standard stock solutions of curcumin, capsaicin, caffeine, and the internal standard (IS)

1.Preparation of standard curcumin, capsaicin, caffeine and internal standard (IS) stock solutions

Curcumin, capsaicin, and caffeine standards were accurately weighed and dissolved with ethanol into a 10 ml volumetric flask. Finally, the concentration of each standard stock solution was 1,000 μg/ml. For the IS stock solution, methyl red was accurately weighed and dissolved with ethanol. Further dilution was made to obtain the final working concentration of 150 μg/ml.

2.Preparation of working standard solutions and HPLC method validation

Curcumin, capsaicin, and caffeine standard stock solutions were accurately pipetted into the same 10.0 ml volumetric flask with ethanol. Serial dilution was performed to yield working standard solutions for each compound in the range of 6.25-150.0 μg/ml. Methyl red was selected as an IS solution. A stock solution of IS was prepared and diluted in ethanol, resulting in a concentration of 150 μg/ml. A 500 μl of IS was pipetted into each working standard solution at 1:1 ratio, yielding a final concentration of 75 μg/ml. The HPLC analytical method for determination for curcumin, capsaicin and caffeine stability was validated according to international standard guidelines. Specificity, accuracy, precision, linearity, limit of detection and quantitation (LOD, LOQ) were evaluated.

3.Preparation of sample solutions for stability determination of curcumin and capsaicin

Approximately 40 mg of turmeric, chili and coffee extracts were accurately weighed. After that, each extract was separately transferred, dissolved and vortex-mixed in ethanol in a 10 ml volumetric flask. The solutions were filtered through a 0.22 μm nylon Acrodisc® filter (Pall, New York, USA) and mixed with the internal standard solution at 1:1 ratio before injection into the HPLC chromatograph. Quantitative measurement of active markers for each extract was performed by comparison with the standard solutions.

Supplementary Material S4. Intra-day and inter-day accuracy and precision results for curcumin, capsaicin and caffeine. Results are expressed as mean ± SD.

Nominal concentration (µg/ml)	Intra-day			Inter-day		
	Curcumin	Capsaicin	Caffeine	Curcumin	Capsaicin	Caffeine
	Recovery (%)					
6.25	105.71±0.80	106.39±1.18	105.97±0.04	103.48±0.11	109.04±2.04	90.25±2.66
12.5	105.20±0.67	101.85±0.99	110.28±1.56	99.60±1.73	91.85±1.79	98.20±1.47
25.0	102.49±1.50	100.39±0.35	105.08±1.02	93.74±1.27	92.70±2.32	92.80±2.66
50.0	99.70±0.13	94.94±1.30	107.91±1.78	92.77±2.27	97.89±1.52	103.29±2.54
75.0	94.18±2.39	89.62±1.10	97.82±1.73	99.24±1.11	92.55±1.34	90.80±2.34
100.0	100.30±0.08	100.36±0.21	106.76±2.63	96.07±3.53	109.90±1.02	94.98±2.32
150.0	100.05±1.89	100.39±0.40	101.98±2.70	97.44±0.68	97.82±0.15	97.44±1.73
Nominal concentration (µg/ml)	Precision (% RSD)					
6.25	0.76	1.11	0.04	0.10	1.88	2.95
12.5	0.64	0.98	3.23	1.74	1.95	1.50
25.0	1.47	0.35	0.97	1.36	2.50	2.87
50.0	0.13	1.37	1.66	2.45	1.55	2.46
75.0	2.55	1.23	1.78	1.13	1.46	2.58
100.0	0.08	0.21	2.47	3.68	0.93	2.44
150.0	1.89	0.41	2.65	0.99	0.16	1.78
Correlation coefficient (r)	0.9976	0.9996	0.9992	0.9913	0.9965	0.9990
Sensitivity						
LLOD (µg/ml)	0.52	0.54	0.40	ND	ND	ND
LLOQ (µg/ml)	6.25	6.25	6.25	ND	ND	ND

Note: ND = not determined

Supplementary Material S 5. Solubility of arabica roasted coffee bean extract, turmeric extract, and chili extract in various vehicles

Vehicle	Solubility		
	Coffee bean extract	Turmeric extract	Chili extract
Avocado oil	Freely soluble	Freely soluble	Slightly soluble
DMSO	Freely soluble	Freely soluble	Soluble
Ethanol	Soluble	Sparingly soluble	Sparingly soluble
Methanol	Freely soluble	Sparingly soluble	Soluble
Glycerin	Slightly soluble	Slightly soluble	Slightly soluble
PEG 400	Freely soluble	Freely soluble	Slightly soluble
PG	Slightly soluble	Sparingly soluble	Slightly soluble
10% Tween® 80	Soluble	Soluble	Slightly soluble
Water	Slightly soluble	Very slightly soluble	Slightly soluble

Supplementary Material S6. Compositions of pre-emulsion formation

Ingredients	Formulations (%w/w)											
	F1	F2	F3	F4	F5	F6	F7	F8	F9	F10	F11	F12
1. Oil mixture	21	21	21	21	21	21	21	21	21	21	21	21
2. Polyglyceryl-3 rice branate	1	2	3	4	5	6	-	-	-	-	-	-
3. Polyacrylamide (and) C13-14 Isoparaffin (and) Laureth-7	-	-	-	-	-	-	1	2	3	4	5	6
4. Polyethylene glycol 400	5	5	5	5	5	5	5	5	5	5	5	5
5.Phenoxyethanol	1	1	1	1	1	1	1	1	1	1	1	1
6. Water	q.s.100	q.s 100	q.s 100	q.s 100	q.s 100	q.s 100	q.s 100	q.s 100	q.s 100	q.s 100	q.s 100	q.s 100

Supplementary Material S7. Physical appearance, pH, mean particle size, PDI, and zeta potential of the selected unloaded nanoemulsions (F4). Different letters (a, b) indicate significant differences among samples at $P < 0.05$

Parameter	F4		
	Baseline	After heating and cooling	After room temperature 2 months
Physical appearance	white color, homogeneous and smooth texture		
pH	6.0		
Particle size (nm)	157.53±1.48[a]	164.53±3.72[a]	169.1±4.94[a]
PDI	0.15±0.02[a]	0.15±0.05[a]	0.22±0.07[a]
Zeta potential (mV)	-44.56±3.45[a]	-50.34±0.74[a]	-41.73±7.93[a]

Supplementary Material S8. the elimination rate constants (k) of curcumin and capsaicin

Samples	Elimination Rate Constant (k) of Curcumin (h^{-1})	Elimination Rate Constant (k) of Capsaicin (h^{-1})
1.Turmeric extract (TE)	$490 \times 10^{-3} \pm 0.00$	$350 \times 10^{-3} \pm 0.00$
2. Turmeric-chili mixture (TCE)	$350 \times 10^{-3} \pm 0.00$	$150 \times 10^{-3} \pm 0.00$
3. Turmeric-chili-coffee mixture (TCE-C)	$300 \times 10^{-3} \pm 0.00$	$170 \times 10^{-3} \pm 0.00$
4. Turmeric-chili mixture with vitamin E (TCE-E)	$250 \times 10^{-3} \pm 0.00$	$120 \times 10^{-3} \pm 0.00$
5. Turmeric-chili mixture with avocado oil (TCE-A)	$230 \times 10^{-3} \pm 0.00$	$120 \times 10^{-3} \pm 0.00$
6. Turmeric-chili-coffee mixture in nanoemulsions (TCE-C-NE)	$120 \times 10^{-3} \pm 0.00$	$90 \times 10^{-3} \pm 0.00$
